# The J-shaped curve: fact or fiction?

**Published:** 2010

## Introduction

Current conventional wisdom suggests that when it comes to good blood pressure control, the lower the better. However, because a pressure of zero over zero is incompatible with life, there would appear to be a J-shaped curve and therefore there must be a point at which lower is not necessarily better.

‘This implies that we need to identify the nadir of that curve to ensure that we don’t inadvertently increase risk when lowering blood pressure by intervening too intensively’, said Dr Meredith. He was addressing a meeting at the South African Heart Association’s annual conference at Sun City in August.

Most blood pressure treatment is guided by epidemiological data that suggest that for every 20-mmHg rise in systolic blood pressure, there is a doubling of cardiovascular mortality risk. Dr Meredith feels that too much of current practice is guided by data that might well be inappropriate. ‘We can’t be certain of the therapeutic implications and it requires a leap of faith to translate epidemiology into treatment, especially in the case of blood pressure that is not especially high to begin with. Matters are complicated by the fact that patients are not homogeneous. Heart failure patients, for example, are not the same as hypertensives and in the context of heart failure, higher blood pressure is indicative of better cardiac function. A cautious approach is therefore needed in these patients, and all factors need to be monitored.’

Dr Meredith cited a number of studies, but underscored their limitations. ‘We really need outcomes trials, of which there are a relative paucity. As a result, much of what we think we know is based on retrospective interpretations of existing trials.

In a 1987 study, Cruickshank argued that the J-shaped curve was more apparent in ischaemic heart disease. In the INVEST study, Messerli found that a diastolic blood pressure lower than 70 mmHg was associated with an increased risk of myocardial infarction, an interesting but far-from-definitive finding.

Sleight’s analysis of the ONTARGET trial showed that while higher blood pressure was associated with a linearly higher stroke risk, when it came to composite outcomes (including myocardial infarction and cardiovascular mortality), there was virtual equivalence between those with the highest and lowest blood pressures, seemingly supporting the idea of the J-shaped curve.’

Dr Meredith was directly involved in a similar post-hoc analysis of the ACTION trial, the first ever randomised, placebocontrolled clinical trial of an anti-anginal drug, nifedipine XL. The study’s overall finding was that blood pressure is an important risk factor in angina patients and that treatment with nifedipine was beneficial.

While Dr Meredith’s findings in respect of stroke echoed Sleight’s on ONTARGET, this was not the case with regard to the other endpoints where the lowest blood pressure was consistently associated with the smallest risk. ‘The prognostic value of baseline systolic and diastolic blood pressure was such that lowest risk was equated with lowest pressure, a different finding from both INVEST and ONTARGET.’

Turning to cardiovascular outcomes with more- versus less-intensive blood pressure lowering, Dr Meredith stated that there were relatively consistent findings in favour of the former, especially in highrisk patients. ‘Intensively treated diabetic patients in the ACCORD study, for example, suffered no deleterious effects and all their outcomes showed a trend in a favourable direction.’

He feels that, in the main, researchers are too obsessed with p-values and that statistical significance may not necessarily translate into therapeutic benefit. ‘I am firmly in the camp that believes lower to be better. Nothing suggests that this is bad for the patient, when therapy is tailored to the individual needs of the patient in question.’

He argued too that in the older placebo-controlled trials that seem to show a J-shaped curve, the increased risk of events was not related to antihypertensive treatment, but rather to poor health conditions at baseline. ‘Our evidence from ACTION suggests that there is no J-shaped curve. Neither is there a nifedipine-induced curve. That said, I acknowledge that there are limitations to our findings, but the same is true for all the other analyses.’

He concluded by advising that some caution should be exercised in accommodating the individual needs of patients. ‘However, failure to control blood pressure remains a major problem and, instead of getting side-tracked and enmeshed in debates about the J-shaped curve, we need rather to focus our energies on achieving good control.’

